# Differences in peripheral myelin antigen-specific T cell responses and T memory subsets in atypical versus typical CIDP

**DOI:** 10.1186/s12883-017-0860-z

**Published:** 2017-04-26

**Authors:** M. Staudt, J. M. Diederich, C. Meisel, A. Meisel, J. Klehmet

**Affiliations:** 1 Department of Neurology, Charité University Medicine, Charitéplatz 1, 10117 Berlin, Germany; 2 Department of Clinical Immunology, Charité University Medicine, Charitéplatz 1, Berlin, Germany

**Keywords:** Chronic inflammatory demyelinating polyneuropathy, T memory subsets, MBP protein, P0 protein, Atypical, Typical

## Abstract

**Background:**

Chronic inflammatory demyelinating polyneuropathy (CIDP) is presented by a large heterogeneity of clinical phenotypes. Around 50% of patients suffer from typical CIDP and show better therapy response than atypical variants. The goal of our study was to search for cellular immunological differences in typical versus atypical CIDP in comparison to controls.

**Methods:**

We evaluated 26 (9 typical, 17 atypical) patients with mainly active-unstable CIDP using clinical and immunological examinations (enzyme-linked immunospot assay ELISPOT, fluorescence-activated cell sorting FACS) in comparison to 28 healthy, age-matched controls (HC). Typical or atypical CIDP measurements were compared with HC using Kruskal-Wallis test.

**Results:**

Atypical CIDP patients showed increased frequencies of T cell subsets, especially CD4+ effector memory T cells (TEM) and CD4+ central memory T cells (TCM) as well as a tendency of higher T cell responses against the peripheral myelin antigens of PMP-22, P2, P0 and MBP peptides compared to typical CIDP. Searching for novel auto-antigens, we found that T cell responses against P0 180-199 as well as MBP 82-100 were significantly elevated in atypical CIDP patients vs. HC.

**Conclusions:**

Our results indicate differences in underlying T cell responses between atypical and typical CIDP characterized by a higher peripheral myelin antigen-specific T cell responses as well as a specific altered CD4+ memory compartment in atypical CIDP. Larger multi-center studies study are warranted in order to characterize T cell auto-reactivity in atypical CIDP subgroups in order to establish immunological markers as a diagnostic tool.

## Background

Chronic inflammatory demyelinating polyneuropathy (CIDP) is the most common autoimmune peripheral neuropathy but remains a rare disease with a prevalence of 0.8-8.9 cases per 100.000 [1, 2]. The disorder causes severe disability in more than 50% of the patients in a chronic-progressive course [1]. Diagnosis can be difficult given the heterogeneity of CIDP phenotypes. About 50% of the patients suffer from so-called atypical variants including *Distal Acquired Demyelinating Polyneuropathy* (DADS) in 25-35% of the cases, *Multifocal Acquired Demyelinating Sensory And Motor Polyneuropathy* (MADSAM) in 15% and rare variants such as pure sensory CIDP (10-13%), pure motor CIDP (<10%) and focal CIDP (2%) [3]. These CIDP subtypes are likely to differ with respect to underlying pathomechanisms and may necessitate different treatment approaches.

Despite recent progress, the underlying immunopathogenetic mechanisms remain poorly understood [4]. Both humoral as well as cellular immune responses are likely to play a role in the induction of autoimmune neuroinflammation, which leads to demyelination and axonal degeneration [4–7].

Peripheral myelin antigens are promising auto-antigens in CIDP pathogenesis. Recently, we demonstrated higher frequencies of auto-reactive IFN-γ responses directed against the peripheral myelin antigens PMP-22 and P2 in treatment naïve patients who responded subsequently well to intravenous immunoglobulin (IVIG) treatment. Clinical improvement under IVIG-treatment correlated with the reduction of antigen-specific responses against PMP-22 and P2 [8].

Experimental studies in the EAN model of Guillain-Barré-Syndrom (GBS) support a pathogenic role of another compact myelin P0. Immunization with P0 180-199 is capable to induce EAN in wildtype-, IFN-γ *knockout* and TNF-α *knockout* mice [9–11]. However, an evaluation in CIDP patients remains to be done.

Myelin basic protein (MBP) is a major constituent of the myelin sheath in the central and peripheral nervous system [12]. Whereas it has been established as an immunodominant auto-antigen for demyelination in the immunopathogenesis of Multiple Sclerosis (MS) its auto-reactive potential in CIDP remains elusive [13].

T cells can be differentiated into CD45RA+ CCR7+ naïve, CD45RA- CCR7- effector memory (TEM), CD45RA- CCR7+ central memory (TCM) and CD45RA+ CCR7-terminally differentiated effector memory (TEMRA) T cells [14]. Especially CD4+ T cells play a major role in CIDP immunopathogenesis [15–17]. In blood and CSF of CIDP patients, significantly elevated frequencies of CD4+ TEM and CD4+ TCM were demonstrated, whereas long-term treated CIDP patients showed significantly reduced CD4+ memory subsets in contrast to untreated CIDP patients [17–19].

Here, we hypothesize that autoreactive myelin-specific T cell responses as well as T cell memory subsets differ between atypical and typical manifestations of CIDP.

## Methods

### Patients

We evaluated 26 CIDP patients using clinical and immunological (enzyme-linked immunospot assay ELISPOT, fluorescence-activated cell sorting FACS) examinations in comparison to 28 healthy, age-matched controls. CIDP patients who met the diagnostic criteria of European Federation of Neurological Sciences (EFNS) 2010 were divided into “typical” vs. “atypical” according to EFNS 2010 [20]. Therapy response was defined as an improvement of ≥2 in Medical Research Council (MRC) sum score in 2 different muscle groups, an improvement of ≥1 in Inflammatory Neuropathy Cause and Treatment (INCAT) score (excluding changes in arm function from 0 to 1) or alternatively an improvement of ≥50% of the walking distance as described previously [8]. Patients and controls were recruited in the outpatient clinic of the Department of Neurology, Charité University Medicine Berlin.

### Peripheral myelin antigens

ELISPOT assay was performed using peptides of seven peripheral myelin antigens and CEF as positive control for T cell responses (Table 1). CEF is a peptide pool containing 23 MCH class 1 restricted viral antigens [21]. Peripheral myelin antigens were provided by Dr. R. Volkmer, Institute of Medical Immunology, Charité University Medicine Berlin. CEF was provided by JPT Peptide Technologies GmbH, Berlin.

**Table 1:** ELISPOT-antigens

antigen	Sequence
PMP-22 32–51	NGHATDLWQNCSTSSSGNVH
PMP-22 51–64	HHCFSSSPNEWLQS
PMP-22120–133	RHPEWHLNSDYSYG
P2 14–25	ENFDDYMKALGV
P2 61–70	EISFKLGQEF
P0 180-199	ASKRGRQTPVLYAMLDHSRS
MBP 82-100	DENPVVHFFKNIVTPRTPP
CEF	peptide pool

### Cryopreservation of Peripheral Blood Monocytes (PBMC)

To evaluate T cell responses efficiently we preserved PBMC in liquid nitrogen over a maximum of 6 months. Blood was sampled in CPT tubes for ELISPOT and in EDTA tubes for flow cytometry. PBMC were isolated within 2 h after venipuncture by 1500 g centrifugation for 20 min. After washing, we diluted the PBMC at a concentration of 2x10^7^cells/ml in freezing medium A (60% FCS; 40% RPMI, Biochrom, Berlin, Germany) at 4 °C. The same volume of freezing medium B (20% DMSO, 80% FCS) at 4 °C was added before cell suspensions were transferred into cryovials (Sarstedt, Nürnbrecht, Germany) and set in one at 4 °C prechilled Nalgene Cryogenic Freezing Container (Fisher Scientific, Hannover, Germany) which was placed in −80 °C overnight. After 12-24 h, cryovials were transferred into liquid nitrogen tanks for storage until ELISPOT.

Thawed cell suspensions were transferred into a 15 ml tube containing 10 ml of ice cold PBS. After two washing steps, cells were pipetted in complete RPMI medium (93% RPMI-1640. 5% heat-inactivated FCS, 1% L-glutamin, 1% penicillin-streptomycin) and counted manually using Trypan blue-staining and light microscopy.

### ELISPOT

IFN-γ ELISPOT assay in this study was performed on human PBMC as previously described [8]. We plated 4×10^5^cells/well in triplicates for each antigen and positive (CEF) or negative control (medium). CEF, a peptide pool containing viral antigens functioning as a positive control for T cell responses, was added at 9 μg/ml [21]. The peripheral myelin antigens PMP-22 32-51, PMP-22 51-64, PMP-22 120-133, P2 14-25, P2 61-70 were used at 40 μg/ml and P0 180-199, MBP 82-100 were used at 20 μg/ml. Spot counts were analyzed via ELISPOT Reader Immunospot (CTL Analyzers, Cleveland, Ohio, USA) and custom software. Spot forming units (SFU) for each antigen were subtracted by SFU of spontaneous IFN-γ secretion (usually <5) and then calculated for a cell amount of 10^6^ cells.

### FACS

Flow cytometry analyses were performed on lymphocyte- and T cell-subpopulations in EDTA whole blood within 12 h after venipuncture.

Flow cytometric analysis was performed as we described recently [17]. Briefly, mouse anti-human fluorescently labelled monoclonal antibodies allowed to quantifying the frequencies of lymphocyte and T cell subpopulations. The following antibodies were used: CD3 Allophycocyanine-Alexa Fluor 750 (APC-A750), CD4 energy coupled dye (ECD), CD8 APC, CD14 Fluorescein isothiocyanate (FITC), CD16 Phycoerythrine (PE), CD19 PE-Cy5.5, CD56 PE, CD45RA Pacific-Blue (PB), CD45 Krome-Orange (KrO) (all by Beckman Coulter) and CCR7 Phycoerythrine (PE) (R&D Systems). Stained samples were evaluated on a ten-colour Navios flow cytometer and were analyzed using Navios Software (Beckman Coulter).

### Statistics

All statistical tests were performed using GraphPadPrism 6.0 software. The study was assessed as an exploratory analysis. Typical or atypical CIDP measurements were compared with healthy, age-matched controls (HC) using Kruskal-Wallis test followed by post-hoc unpaired *t*-test or Mann-Whitney-test when *p* < 0.05. For group differences with regard to sex, prior treatment, disease activity and therapy response, Fisher’s exact test was used. For age and INCAT score, unpaired *t*-test was used. For time since diagnosis, Mann-Whitney-test was used. Level of significance was defined as *p* < 0.05 for all comparative tests.

## Results

### Clinical characterization of typical and atypical CIDP patient group (Table 2)

We recruited 17 (65.4%) male and nine (35.6%) female patients. Mean age was 59 years (range 32-78). 20/26 (76.9%) patients were included in active-unstable stages of the disease, 1 (3.8%) with active-stable CIDP and five (19.2%) in clinical remission [22]. 12 (46.2%) patients were treatment naïve whereas 10 (38.5%) received IVIG therapy and four (15.4%) glucocorticosteroids (GS) prior to our study. We classified 9 (38.5%) as typical and 17 (61.5%) as atypical CIDP patients, including 6 with pure sensory CIDP, 4 with MADSAM, 5 with DADS, 1 with pure motor CIDP and 1 with a sensory-ataxic course of disease.

**Table 2:** Clinical information (*n* = 26; IVIG intravenous immunoglobulins, CIDP chronic inflammatory demyelinating polyneuropathy, INCAT Inflammatory Neuropathy Cause and Treatment, GS glucocorticosteroids)

		typical	atypical	*p*-values atypical vs. typical
Sex	male	3/9 (33%)	14/17 (82%)	0.013
	female	6/9 (66%)	3/17 (18%)	
Age (years)	mean	61.0	57.2	0.512
	range	32-78	33-74	
Previous treatment	None	3/9 (33%)	9/17 (53%)	0.429^a^
	IVIG	4/9 (44%)	6/17 (35%)	0.652^a^
	Steroid	2/9 (22%)	2/17 (12%)	0.547^a^
Time since diagnosis	mean	4.1	4.2	0.860
(years)	range	1-8	1-7	
INCAT	mean	3.2	2.3	0.045
	Range	<1-8	<1-7	
Disease activity	range	1-5	1-3	
	active-unstable	6/9 (66%)	14/17 (82%)	1.000^b^
	active-stable	1/9 (11%)	0/17 (0%)	0.333^b^
	in remission	2/9 (22%)	3/17 (18%)	1.000^b^
Therapy response	responder	9/9 (100%)	8/17 (47%)	0.022
	non-responder	0/9 (0%)	9/17 (53%)	

Fishers exact test for sex, previous treatment, disease activity and therapy response

^a^compared versus treatment naïve patients

^b^compared versus remission state; unpaired *t*-test for age and INCAT score; Mann-Whitney test for time since diagnosis

Therapy-responders were classified as 9/9 (100%) typical and only 8/17 (47%) atypical CIDP patients. As controls we used age-matched, healthy patients (HC). For ELISPOT-analyses 14 HC (mean age 70, range 53-83) and for FACS-analyses 28 HC (mean age 61, range 42-83) were evaluated.

### T cell IFN-γ- responses to P0 180-199 and MBP 82-100 were elevated in CIDP patients compared to healthy controls

T cell responses against the peripheral myelin antigens, P0 180-199 and MBP 82-100 were measured by IFN-γ ELISPOT in a cohort of 26 CIDP patients. Due to spontaneous IFN-γ-production, 6 patients (1 typical, 5 atypical) were excluded for further ELISPOT analysis.

**Fig. 1:**
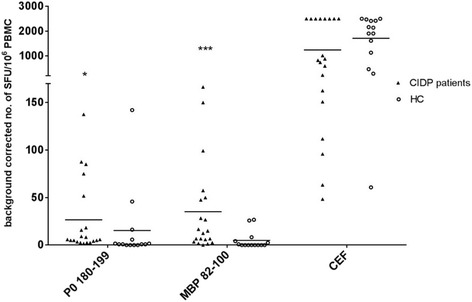
Frequency of P0 and MBP specific T cells in CIDP patients. Frequencies of peripheral myelin antigen-specific T cell responses in CIDP patients (*n* = 20) vs. HC (*n* = 14) measured by IFN-y ELISPOT. Background corrected SFU per 10^6^ PBMC were significantly elevated for P0 180-199 as well as MBP 82-100 in CIDP patients vs. HC. Maximum value defined due to methodical limitations (CEF = 2500). (*p < 0.05, **p < 0.01, ***p < 0.001). Scatter dot plot with line at mean

T cell responses against P0 180-199 as well as MBP 82-100 were significantly elevated in CIDP patients vs. HC: P0 180-199 (*p* < 0.05), MBP 82-100 (*p* < 0.001) (Fig. 1). CEF-specific IFN-γ-production in CIDP did not differ from HC excluding unspecific T cell activation in CIDP.

### Increased myelin antigen-specific T cell responses in atypical CIDP

**Fig. 2:**
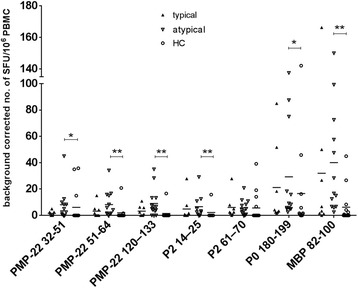
Frequencies of peripheral myelin antigen-specific T cell responses in typical versus atypical CIDP patients. Typical (*n* = 8) vs atypical CIDP patients (*n* = 12) vs. HC (*n* = 14) were measured by IFN-y ELISPOT. Background corrected SFU per 10^6^ PBMC. Significantly, elevated SFU were observed in atypical CIDP patients vs. HC for PMP-22 32-51, PMP-22 51-64, PMP-22 120-133, P2 14-25, P0 180-199, MBP 82-100. (*p < 0.05, **p < 0.01, ***p < 0.001). Scatter dot plot with line at mean. For P0 180-199, a cut-off value of 5 SFU/10^6^ PBMC in T cell-Elispot having a sensitivity of 91,7% (11/12) and a specificity of 62,5% (5/8) with AUC 0.69. For MBP 82-100 using a cut-off value of 10 SFU per 10^6^ PBMC in a T cell-Elispot assay had a sensitivity of 75,0% (9/12) and a specificity of 62,5% (5/8) with AUC 0.68

Atypical CIDP variants tended to have increased IFN-γ responses to all 7 tested peripheral myelin antigens compared to both, typical CIDP patients and HC (Fig. 2). This difference between typical and atypical CIDP patients was more pronounced for PMP-22 32-51 (*p* = 0.0621), PMP-22 51-64 (*p* = 0.1050), PMP-22 120-130 (*p* = 0.1451), P0 180-199 (*p* = 0.1894) and MBP 82-100 (*p* = 0.1841) (Fig. 2). In comparison to HC, atypical CIDP patients showed significantly higher SFU for the following peripheral myelin antigens: PMP-22 32-51 (p_atypical_ < 0.05), PMP-22 51-64 (p_atypical_ < 0.01), PMP-22 120-130 (p_atypical_ < 0.01), P2 14-25 (p_atypical_ < 0.01), P0 180-199 (p_atypical_ < 0.05), MBP 82-100 (p_atypical_ < 0.01) (Fig. 2). CEF responses did not differ between tested groups.

### Atypical CIDP variants have significantly higher levels of Cd4+ memory T cells

**Fig. 3:**
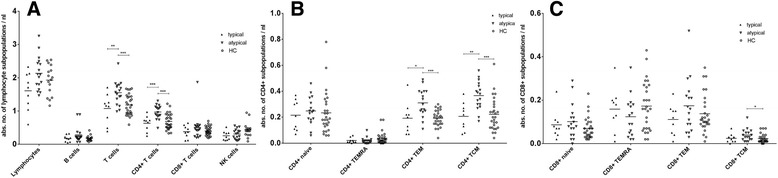
Quantitative analysis of lymphocyte subpopulations in typical versus atypical CIDP. Lymphocyte subpopulations of typical (*n* = 9) vs. atypical CIDP patients (*n* = 17) vs. HC (*n* = 28) were measured by flow cytometry. In atypical CIDP patients significantly higher frequencies of T cells and CD4+ T cells were seen compared to vs. typical CIDP patients and HC (**a**). Significantly higher frequencies for CD4+ TEM and TCM in atypical vs. typical CIDP patients and HC (**b**). Significantly higher frequencies for CD8+ TCM in atypical CIDP patients vs. HC (**c**). (*p < 0.05, **p < 0.01, ***p < 0.001). Scatter dot plot with line at mean

Frequencies of T cells (*p* < 0.01) and CD4+ T cells (*p* < 0.001) were higher in patients with atypical CIDP variants in comparison to typical CIDP patients (Fig. 3a).

Investigating CD4+ T cell subpopulations, CD4+ memory T cell subsets were significantly increased in atypical vs. typical CIDP patients, as shown for CD4+ TEM (*p* < 0.05) and CD4+ TCM (*p* < 0.01) in Fig. 3b.

Likewise CD8+ TEM (*p* = 0.1745) and CD8+ TCM (*p* = 0.1475) tended to be increased in atypical compared to typical CIDP patients. Further compared to HC, atypical CIDP patients had significantly elevated CD8+ TCM-frequencies (*p* < 0.05) (Fig. 3c).

## Discussion

In the present study, typical CIDP differed from the group of atypical variants. Here, we found a stronger activated immune system in patients suffering from atypical variants of CIDP defined by a trend towards increased peripheral myelin antigen-specific (PMP-22, P0 180-199, MBP 82-100) T cell responses associated with a specific altered CD4+ memory compartment of increased CD4+ TEM and CD4+ TCM counts in the blood. Further we detected elevated T cell responses against antigens P0 180-199 and MBP 82-100 in CIDP patients which have not described before.

We confirmed or previous findings that changes of the T memory compartment is a common finding especially in untreated patients [8, 17], which is in contrast to Sanvito and colleagues who showed no differences in T cell subpopulation [23]. In the present study, we detected elevated TEM and TCM primarily in atypical CIDP patients. Clinical experience and studies suggest that typical CIDP patients respond better to therapy than atypical CIDP variants, especially DADS [24], which is in line with our presented data showing that 100% (9/9) of typical compared to 47% (8/17) of atypical CIDP patients were therapy-responders. The reason for different treatment responses of CIDP subtypes remains unknown. The higher specific immune responses against myelin-derived peptides in atypical compared to typical variants may be a cause for the lower treatment-responses. Likewise, the increased immune reactivity in atypical CIDP patients could result from insufficient treatment.

Recently, it has been demonstrated that CIDP patients show a diminished pro-regenerative function of Schwann cells leading to the axonal loss and therefore incomplete clinical recovery after treatment which is probably caused by inflammatory mediators [25]. Thus, differences in immune responses between typical and atypical CIDP we have demonstrated might also influence Schwann cell function resulting in different treatment responses and long-term outcome. The INCAT score was significantly lower in atypical cases. However, there was no difference in the time since diagnosis so that a longer disease course and hence pronounced disability and/or altered immune response is not the cause of this difference. Yet, we included mainly atypical case with mild motoric disability (6 patients with sensory CIDP [35.3%] and 5 patients with DADS [29.4%]) who are less often dependent on walking aids leading to lower INCAT disability scores.

Since we included mainly clinically unstable patients who had partly received treatment before, we are not able to answer this question at present. Based on previous results of reduced CD4+ memory subsets in GS-treated patients [17], it might be further argued that GS treatment may be efficient for this patient group. In contrast to Sanvito et al. [26], we identified higher IFN-γ responses to P2 and PMP22 peptides which have been more pronounced in the atypical compared to the typical CIDP subgroup. A higher number and proportion of atypical patients might explain this discrepancy as well as the fact that we included mainly clinically unstable and newly diagnosed patients.

Earlier publications detected P0 IgG-antibodies in CIDP patients and P0 180-199 specific T cell responses in spontaneous autoimmune polyneuropathy-mice [11]. Although EAN resembles Guillain-Barré-Syndrom (GBS) much more than CIDP, we regarded P0 as possible further candidate autoantigen of compact myelin for CIDP. Here, we detected elevated P0 180-199 specific T cell responses primarily in atypical CIDP.

Up to now, only little is known about the role of MBP 82-100 in the pathogenesis of CIDP even though MBP has been detected as part of the myelin sheath of peripheral nerves. Nevertheless, there is long-standing evidence that MBP 82-100 can induce neuroinflammation in autoimmune diseases [27]. Glatirameracetat, known antagonist of MBP 82-100 specific T cell receptor and part of MS therapy has been demonstrated to alleviate symptoms also in EAN- mice [28, 29]. Here, we demonstrated significantly elevated MBP 82-100 specific T cell responses in CIDP patients, again primarily in patients with atypical manifestations.

There is growing evidence for the autoimmune potential of antigens which are derived from non-compact myelin of the nodal/paranodal region such as neurofascin 155 of 186 leading to antibody response in distinct subgroups of CIDP or multifocal motoric neuropathy (MMN) [30–33]. Thus, antigenic targets derived from both compact and non- compact myelin leading to humeral and/or cellular immune response may define underlying immune mechanism of different clinical phenotypes of CIDP.

Several limitations may have affected our results. First, our clinically heterogeneous group of atypical patients was too small to distinguish between subgroups of atypical CIDP, which would be necessary to characterize atypical subtypes and to define specific cut offs for our immunological parameters. Second, differences in gender and INCAT score between typical and atypical CIDP patients might have influenced our immunological findings. Third, we aimed to recruit treatment-naïve patients in active-unstable stages of the disease. However, only 46% of patients (12/26) were treatment-naïve at enrollment. Previous immunosuppressive and –modulating therapy might have influenced our immunological findings.

## Conclusions

Higher myelin-antigen specific T cell responses together with elevated T cell memory subsets were found in atypical compared to typical CIDP patients suggesting different patterns of immune responses in clinically distinctive CIDP subgroups. Myelin as well as nodal/paranodal proteins might serve as candidate autoantigens to establish robust immune markers for CIDP subtype differentiation. Given the clinical diversity of CIDP a larger cohort study is warranted in order to establish those markers with reliable cut-off values.

## Abbreviations

CIDP: Chronic inflammatory demyelinating polyneuropathy
DADS: Distal acquired demyelinating polyneuropathy
EAN: Experimental autoimmune neuritis
EFNS: European Federation of Neurological Sciences
ELISPOT: Enzyme-linked immunospot assay
FACS: Fluorescence-activated cell sorting
GBS: Guillain-Barré-Syndrom
GS: Glucocorticosteroids
HC: Healthy control
IFN-γ: Interferon-gamma
INCAT: Inflammatory neuropathy cause and treatment
IVIG: IV immunoglobulin
MADSAM: Multifocal Acquired Demyelinating Sensory And Motor Polyneuropathy
MBP: Myelin basic protein
MGUS: Monoclonal gammopathy of uncertain significance
MRC: Medical Research Council Scale
MS: Multiple sclerosis
PBMC: Peripheral blood mononuclear cells
SFU: Spot forming unit
TCM: Central memory T cells
TEM: Effector memory T cells
TEMRA: Terminally differentiated T cells
